# Proteogenomic Approaches for the Identification of NF1/Neurofibromin-depleted Estrogen Receptor–positive Breast Cancers for Targeted Treatment

**DOI:** 10.1158/2767-9764.CRC-23-0044

**Published:** 2023-07-26

**Authors:** Beom-Jun Kim, Ze-Yi Zheng, Jonathan T. Lei, Matthew V. Holt, Anran Chen, Jianheng Peng, Diana Fandino, Purba Singh, Hilda Kennedy, Yongchao Dou, María del Rosario Chica-Parrado, Emmanuel Bikorimana, Dan Ye, Yunguan Wang, Ariella B. Hanker, Nada Mohamed, Susan G. Hilsenbeck, Bora Lim, Jaya Ruth Asirvatham, Arun Sreekumar, Bing Zhang, George Miles, Meenakshi Anurag, Matthew J. Ellis, Eric C. Chang

**Affiliations:** 1 Lester and Sue Smith Breast Center, Baylor College of Medicine, Houston, Texas.; 2 Department of Medicine, Baylor College of Medicine, Houston, Texas.; 3 Health Management Center, the First Affiliated Hospital of Chongqing Medical University, Chongqing, P.R. China.; 4 Department of Molecular and Human Genetics, Baylor College of Medicine, Houston, Texas.; 5 Simmons Comprehensive Cancer Center, UT Southwestern Medical Center, Dallas, Texas.; 6 Baylor Scott and White Health, Dallas, Texas.; 7 Department of Molecular and Cellular Biology, Baylor College of Medicine, Houston, Texas.

## Abstract

NF1 is a key tumor suppressor that represses both RAS and estrogen receptor-α (ER) signaling in breast cancer. Blocking both pathways by fulvestrant (F), a selective ER degrader, together with binimetinib (B), a MEK inhibitor, promotes tumor regression in NF1-depleted ER^+^ models. We aimed to establish approaches to determine how NF1 protein levels impact B+F treatment response to improve our ability to identify B+F sensitive tumors. We examined a panel of ER^+^ patient-derived xenograft (PDX) models by DNA and mRNA sequencing and found that more than half of these models carried an *NF1* shallow deletion and generally have low mRNA levels. Consistent with RAS and ER activation, RET and MEK levels in NF1-depleted tumors were elevated when profiled by mass spectrometry (MS) after kinase inhibitor bead pulldown. MS showed that NF1 can also directly and selectively bind to palbociclib-conjugated beads, aiding quantification. An IHC assay was also established to measure NF1, but the MS-based approach was more quantitative. Combined IHC and MS analysis defined a threshold of NF1 protein loss in ER^+^ breast PDX, below which tumors regressed upon treatment with B+F. These results suggest that we now have a MS-verified NF1 IHC assay that can be used for patient selection as a complement to somatic genomic analysis.

**Significance:**

A major challenge for targeting the consequence of tumor suppressor disruption is the accurate assessment of protein functional inactivation. NF1 can repress both RAS and ER signaling, and a ComboMATCH trial is underway to treat the patients with binimetinib and fulvestrant. Herein we report a MS-verified NF1 IHC assay that can determine a threshold for NF1 loss to predict treatment response. These approaches may be used to identify and expand the eligible patient population.

## Introduction

Neurofibromatosis type-1, caused by germline mutations in *NF1*, is the commonest inherited disorder, affecting 1 in 3,000 births (1). Neurofibromatosis patients are predisposed to both benign and malignant tumors of the nervous system, as well as an increased risk for diverse cancers, including breast cancer (2–4). Somatic *NF1* mutations are also frequently detected in sporadic cancers (5, 6). Thus, *NF1* deficiency underlies the formation and/or progression of multiple cancer types beyond the neoplastic manifestations of neurofibromatosis.

*NF1* encodes neurofibromin, whose well-understood function is to repress RAS signaling as a GTPase-activating protein (GAP). The most common form of breast cancer expresses estrogen receptor α (ER^+^), which functions as a ligand-dependent and -independent transcription factor (7). In the presence of estradiol (E2), the ER complex assembles with coactivators on chromatin to activate gene expression. Conversely, corepressors displace coactivators in the presence of tamoxifen, an endocrine treatment that antagonizes ER signaling in breast cancer. Guided by a clinical study seeking mutations associated with a poor response to adjuvant tamoxifen (8), we uncovered a GAP-independent activity of NF1, whereby NF1 binds ER directly to act as a transcriptional corepressor (9). NF1 inactivation alters ER transcriptional properties causing tamoxifen to function as an agonist rather than an antagonist. NF1 loss also promotes proliferation in suboptimal concentrations of E2. These effects explain clinical associations between low *NF1* expression and/or mutation and resistance to both tamoxifen and aromatase inhibition (8, 9). Using a NF1-deficent ER^+^ patient-derived xenograft (PDX) mouse model (WHIM16), we determined that marked tumor regression can be achieved with the combination of fulvestrant (F), which is a pure ER antagonist and degrader, and the MEK inhibitor binimetinib (B), to inhibit RAS signaling downstream of RAF (9). This treatment strategy is being tested in ER^+^ metastatic breast cancer in a phase II clinical trial as part of the NCI ComboMATCH program (NCT05554354).

For targeted therapy, accurate biomarker assessment is essential. In our previous study, *NF1* frameshift (FS) and nonsense (NS) mutations, detectable by whole-exome sequencing (WES), consistently correlated with poor patient outcome after tamoxifen treatment, and we have evidence that these mutations can lead to loss of NF1 protein due to nonsense mRNA decay or protein instability (9). Exactly how closely *NF1* mutations predict protein loss has not been thoroughly studied using clinically relevant samples, and currently there are no protein-based NF1 diagnostics used to guide the endocrine treatment in ER^+^ breast cancer. We hypothesized in this study that *NF1* FS/NS mutation detection alone underestimates the number of patients with NF1-depleted ER^+^ tumors. NF1 loss-of-function mutations are detectable in about 2% of primary ER^+^ breast tumors (8), but up to 20% of the primary ER^+^ breast cancer can be considered NF1-deficient because of an association with a poor response to adjuvant endocrine therapy at the mRNA level (9). If the hypothesis that NF1 loss is more common at the mRNA or protein level is correct, then the majority of patients eligible for B+F treatment will be missed. Therefore, in this study, we took a proteogenomic approach to optimize the determination of NF1 protein levels directly in breast cancer samples.

## Materials and Methods

### Cell Culture and Reagents

MCF-7, T47D, MDA-MB-231, MCF-10A and the cell lines that were derived from these were cultured as described previously (9, 10). OVCA429 cells (a kind gift from Dr. Kwong Wong, MD Anderson Cancer Center, University of Texas, Houston, TX) were cultured in DMEM supplemented with 10% FBS. NF1 loss in OVCA429 cells is associated with a 17 bp deletion in exon-17 as first reported by Sangha and colleagues (11). “NF1-KO” was created by CRISPR-Cas9–mediated knockout (pLentiCRISPRv2-*NF1* #1; ref. 9). MDA-MB-231 cells were transduced by pCL-FLAG-NF1 (9), followed by puromycin (1 μg/mL) selection for 48 hours, to overexpress NF1. The vector control was pCL-FLAG.

### Gene Copy Number and Exon-wise Gene Expression Coverage Determination

For somatic copy-number alteration (CNA) analysis, alignment files WES was processed using CopywriteR package (12) to derive log_2_ tumor-to-normal copy-number ratios. The genome-wide chromosome instability index will then be derived by adding up the instability scores for all 22 autosomes in each sample. GISTIC2 (13) was used to retrieve gene-level copy-number values and call significant CNAs in the cohort. A threshold of ±0.3 was applied to log_2_ copy-number ratio to identify gene-wise gain or loss of copy number, respectively. Mutation identification method has been reported previously (14).

To determine exon-wise expression coverage, RNA sequencing (RNA-seq)-based alignment files were used as input for bamtobed, sort and coverage functions from bedtools package (15) using *NF1* isoform uc002hgg as reference to obtain coverage for coding sequence (CDS) 0–57 of the *NF1* gene. Fraction of bases that had coverage in PDX RNA-seq data relative to CDS were calculated and ranged from 1 to 0.

### Proteomics

The Kinase Inhibitor Pulldown Assay (KIPA) assay was performed as described previously (16). Briefly, nine protein kinase inhibitors (palbociclib, crizotinib, GSK690693, AZD4547, CZC-8004, afatinib, FRAX597, abemaciclib, and axitinib) were conjugated to ECH Sepharose-4B bead, and the resulting bead was used for analyzing the ER^+^ PDX and preoperative letrozole (POL) samples. We routinely prepare a reference protein lysate derived from a panel of cell lines as quality control for KIPA, because all together the mixture contains over 92% of kinases in the human genome (16). To this end, 100 μg protein lysates were used and pulled-down proteins were analyzed by mass spectrometry (MS). NF1 levels were quantified by KIPA-DDA (data-dependent acquisition), while other proteins were quantified by KIPA-PRM (parallel reaction monitoring).

To choose the best responsive peptide for NF1 quantification, 3 most appropriate candidates were selected from peptide information in KIPA experiments (FDEQLPIK, VGSTAVQVTSAER, VAETDYEMETQR). These peptides were tested with the PRM method and FDEQLPIK has been selected as the peptide for SureQuant assay. In Figs. 3D and 5B, palbociclib-conjugated ECH-agarose beads were used for NF1 pulldown and NF1 level was quantified by SureQuant. For quantification, we take the ratio of the endogenous light peptide to that of the stable isotope “heavy” peptide, and multiply by the known amount of heavy peptide (in moles or grams). This gives us the amount in mol of endogenous peptide loaded on the column for mass spectrometry. To normalize loading variations, we divide the amount of endogenous peptide by “total ion chromatogram” (TIC). TIC is the total signal acquired by the MS and represents the summed intensity and total amount of all peptides loaded on the column.

We performed KIPA profiling and analysis using eleven patient samples from a multicenter phase II clinical trial where postmenopausal women with >2 cm, ER^+^ or PR^+^ breast cancer were enrolled in a trial of 16 to 24 weeks of letrozole 2.5 mg daily before operation. The patients were administered with POL study (17). For the POL cohort tumor, tumor content was assessed by a pathologist and only samples with tumor contents at least 50% were included in the analysis. For PDX models, proteins that are of human origin were assessed computationally as described previously (18).

### Protein Gel Electrophoresis and Western Blots

NF1 full-length or truncated proteins pulled down by KIPA beads were loaded to SDS-PAGE gel and separated by electrophoresis. The gel was then strained by Flamingo Fluorescent Gel Stain (Bio-Rad). Cell/tumor lysates were prepared and analyzed by Western blots as described previously (9). The antibodies target NF1 and GAPDH were from MilliporeSigma (MABN2557, Clone NF1-A376G3) and Santa Cruz Biotechnology, respectively. The antibodies against ERα (D8H8), ERK1/2, Phospho-ERK1/2 (T202/Y204), and β-tubulin (9F3) were all from Cell Signaling Technology.

### Sample Preparation for NF1 IHC Assay

Tissue culture cell pellets were created from cells cultured in 10–15 cm dishes to 90% confluence, fixed in 10% neutral buffered formalin (NBF), washed with 10 mmol/L PBS (pH 7.4), and suspended in 4% molten agarose. To avoid variations caused by sample preparation, all PDX tissues were fixed overnight in 10% NBF at room temperature and washed in 70% ethanol. All samples were processed and embedded by standard protocol on a SAKURA Tissue-Tek VIP6 and Tissue Embedder, respectively. Cut tissue sections were (5 μm) placed on charged glass slides and baked at 58°C (10–12 hours) in a dry slide incubator, deparaffinized with xylene and rehydrated via an ethanol step gradient.

The patient-derived organoids (PDO) were generated from a metastatic ER^+^ patient cohort as described previously (19). Two of the PDOs used in this study carry the same *NF1* mutations as found in the matched patient biopsies: PDO-412421 carries NF1^S1329*/S365*^, while PDO-33682 carries a G to C mutation at the end of intron-5 (Ch 17 at position 31181421), which is predicted to disrupt the 3′-splice site leading to exon skipping. The splicing mutation has a very high variant allele frequency of 87.6%. To prepare for IHC, PDOs were fixed in 10% buffered formalin (3 mL per well in a 6-well plate) for 1 hour at 37°C. Careful pipetting up and down was performed to disrupt the Matrigel to release the PDOs. Fixed PDOs were washed and resuspended in 1.5 mL PBS and centrifuged at 6,000 rpm for 2 minutes at 30°C. Supernatant was removed by aspiration, and the PDOs were placed in a heat block at 45°C. PDOs were resuspended in 300 μL of 2.5% low-melt agarose (FMC Nusieve GTG catalog no. 50080) in PBS and centrifuged at 6,000 rpm for 30 seconds. The tube was turned 180° and centrifuged again at 6,000 rpm for 15 seconds to ensure a compact pellet at the apex of the tube. Agarose-embedded organoids were embedded in paraffin and sectioned (5-μm sections) at the UT Southwestern Tissue Management Shared Resource before IHC.

The preparation of patient-derived tumor microarrays (TMA) is an ongoing process as conducted at Baylor Scott and White Health (Institutional Review Board approval number 020-393). The pathology laboratory information system was queried for a biopsy diagnosis of invasive breast carcinoma and subsequent samples of regional and distant metastatic disease. Hematoxylin and eosin (H&E)-stained slides from all available surgical samples of initial biopsy, subsequent breast surgery, lymph node dissection, and biopsies of distant metastases were retrieved from the archives and reviewed by a pathologist with expertise in breast disease. Archived formalin-fixed paraffin blocks were then retrieved and selected foci were manually cored out to construct TMAs with *in situ*, invasive, regional lymph node and distant metastatic cancer. Each TMA thus contained tissues representing cancer progression in a single patient. Both decalcified and non-decalcified samples of tumor in bone were included along with normal breast epithelial internal control when available. A comprehensive analysis of NF1 levels in metastasis will be carried out in the future. Benign corticomedullary renal tissue from a single specimen served as an external control across all patient TMAs. Slides were then cut at 5 μm thickness for H&E-stained slides. Positively charged slides were used for all IHC studies.

### NF1 IHC Assay

Routine peroxidase blocking and heat-induced antigen retrieval were performed with FLEX TRS High pH buffer (Agilent). Primary NF1 antibody (MilliporeSigma, above) incubation was performed per standard protocol (1:250 dilution) at room temperature for 1 hour followed by incubation with Envision Plus Mouse linker and standard chromogenic staining protocol with the Envision Polymer-HRP anti-mouse/3,3′diaminobenzidine (Dako). All slides were counterstained in Harris hematoxylin. IHC analysis was performed and evaluated against positive and negative tissue and cell line controls.

### Animals

All animal described animal experiments were approved by the Baylor College of Medicine Institutional Animal Care and Use Committee. PDX tumors were engrafted into cleared mammary fat pads of SCID/bg female mice (Envigo International) and allowed to grow without exogenous E2 supplementation until tumors reached approximately 200 mm^3^. Mice were then randomized into different treatment groups. Fulvestrant was injected subcutaneously at 250 mg/kg weekly, while binimetinib (20 mg/kg) were given daily as chow (Research Diets Inc.). Tumor size was measured twice weekly, and mouse body weight was also measured weekly to monitor treatment toxicity.

### Statistical Methods

All statistical methods relevant to a particular experiment are already described in the main text and/or in the figure legends. *P* < 0.05 was considered statistically significant.

### Data Availability Statement

The data and reagents generated by the authors are available upon request.

## Results

### Genomic Alterations in the *NF1* Gene in ER^+^ PDXs

**FIGURE 1:**
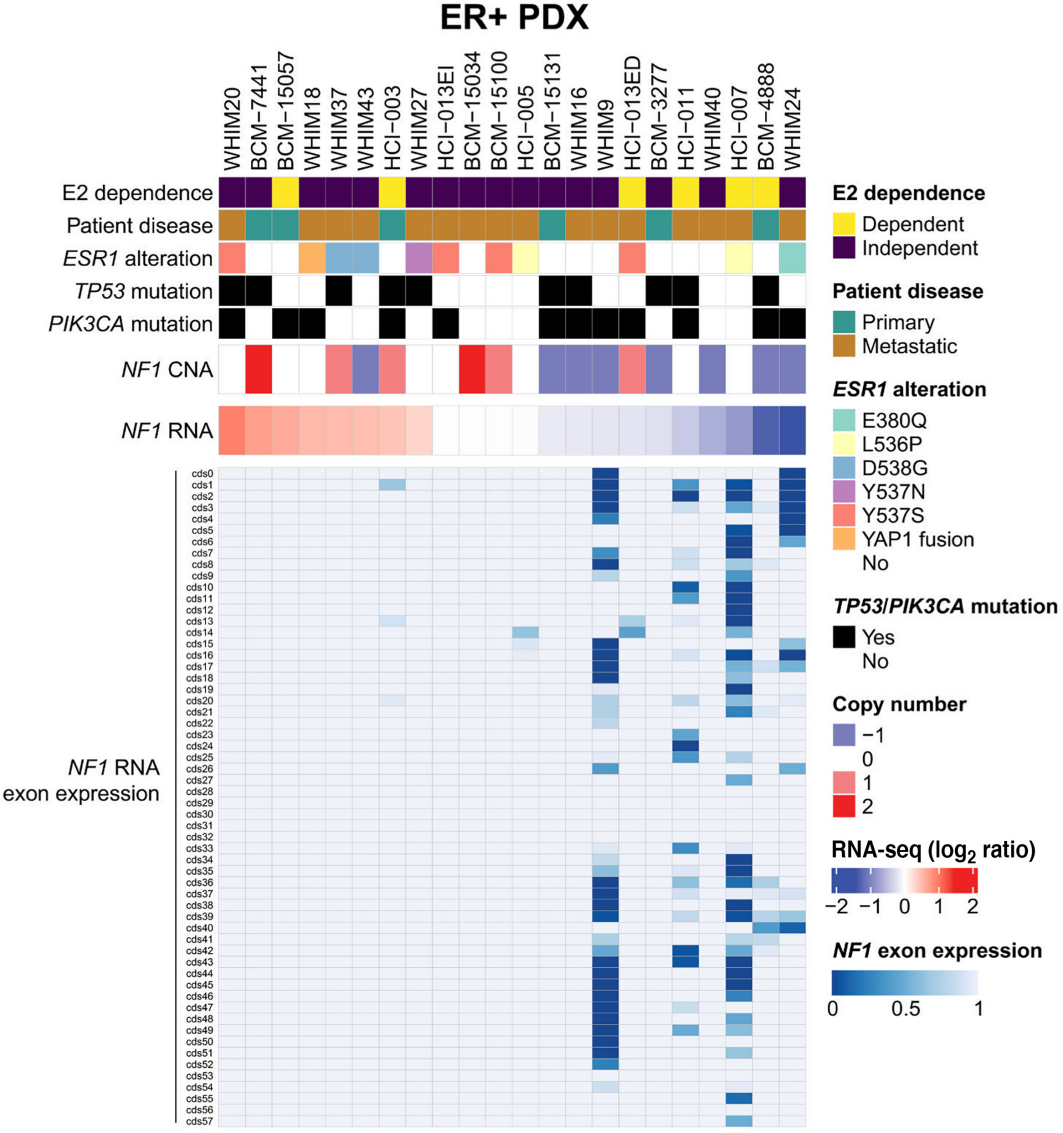
CNA complements mRNA for assessing NF1 status in ER^+^ PDX models. Sample map of 22 ER^+^ PDXs showing E2-dependent growth, patient disease state of sample used to generate the PDX, presence of recurring *ESR1* mutations/fusions, mutation status of *TP53, PIK3CA,* and *NF1*. Also shown is *NF1* CNAs where “0” represents no change in copy number, “–1” as shallow deletion, and “+1” and “+2” low- and high-level amplification, respectively. Full-length *NF1* (NM_001042492) mRNA levels were determined by RNA-seq. The bottom panel shows *NF1* exon expression by RNA-seq across *NF1* CDS in PDX tumors.

To optimize approaches to diagnose NF1-depleted ER^+^ tumors, we examined 22 ER^+^ PDX models (20). Proteogenomic analyses were used to identify molecular features that associate with E2 dependence for tumor growth (14, 16). Here we focused on the proteogenomic status of NF1 across these PDXs (Fig. 1). We have previously used the presence of *NF1* NS and FS mutations by WES to predict NF1 inactivation (8). However, we found only one PDX model (BCM-15131) harbored a FS mutation (*pR2237_K2244del*). In our previous study, *NF1* shallow deletion was also observed in patient samples (8). When we similarly assessed *NF1* copy number alterations (CNAs) in our PDX panel, we found that almost half (*n* = 8) carry single copy loss (Fig. 1, −1 copy). Tumors carrying such shallow deletion in *NF1* showed modest correlation with low mRNA levels, although the correlation was not statistically significant (Supplementary Fig. S1A). Overall, no significant difference was observed between *NF1* expression in + and − E2 conditions (Supplementary Fig. S1A). Deep *NF1* deletion (−2 copies) was not detected in any of our models. However, *NF1* is a very large gene that contains at least 57 exons (Supplementary Fig. S1B). We hypothesized that mutations in *NF1* can be cryptic but detectable based on low mRNA expression (Fig. 1; ref. 21). Consistent with this suggestion, mRNA expression exon by exon was uneven across the *NF1* coding sequence in tumors with shallow deletion and low expression of *NF1,* with the 5′-exons often underexpressed relative to the 3′-exons (Fig. 1).

### A Direct Interaction Between NF1 and Kinase Inhibitor–conjugated Beads is Detected by Protein MS

**FIGURE 2:**
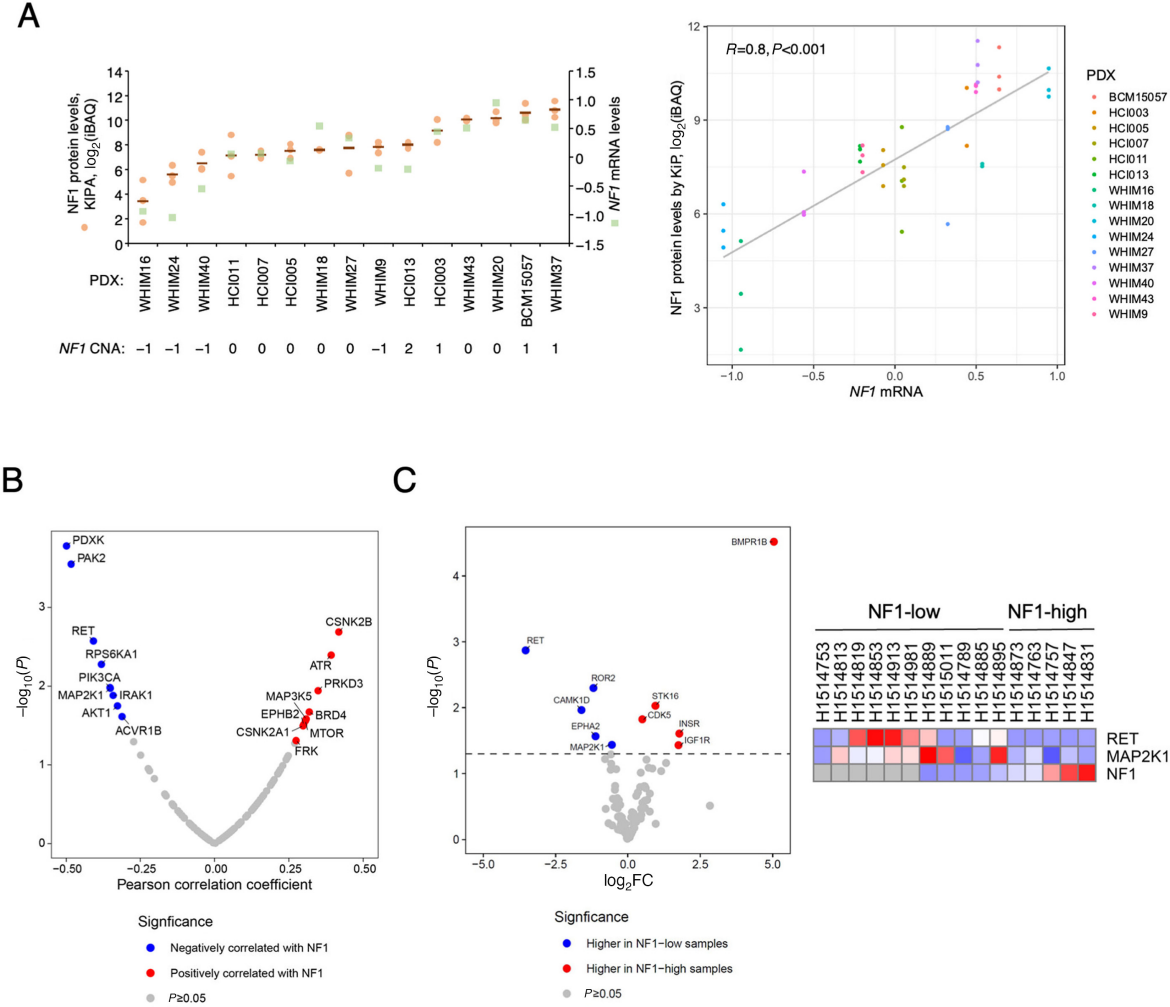
Detection of NF1 by a MS-based approach (KIPA) after pulldown by beads conjugated to kinase inhibitors. **A,***NF1* mRNA was determined by RNA-seq, while NF1 protein levels (iBAQ, the sum of all the peptides intensities divided by the number of theoretical peptides from the protein) was quantified by KIPA. Correlation coefficient and *P* value were determined by Pearson correlation. *NF1* copy-number status was as described in Fig. 1. **B,** Protein correlation with NF1 protein kinase levels as determined by KIPA in PDXs. *P* values and *R* were determined by a linear regression model. **C,** Volcano plot shows differences in protein abundance as log_2_FC (fold change) in baseline POL samples that were stratified into NF1-low versus NF1-high groups. The heat map shows relative levels of indicated proteins. *P* values determined by Student *t* test.

To examine the effects of NF1 loss on protein kinase abundance, we deployed an assay based on a panel of protein kinase inhibitors individually conjugated to sepharose beads to profile the expression of different branches of the kinase family at the protein level. After a tumor lysate pulldown and subsequent wash step to remove low affinity interactions, MS was used to detect protein–bead interactions in an unbiased manner. We refer to our version of this well-established approach the KIPA (16, 22, 23). We created a workflow in which analyses can be carried out using as little as 50–100 μg protein (24). Unexpectedly, we found that NF1 was detected by KIPA (16) in a manner that correlated with *NF1* mRNA levels (correlation coefficient of 0.8, *P* < 0.001; Fig. 2A).

We subsequently determined correlations between NF1 levels detected by KIPA and kinase levels copurified by the drug beads. KIPA data demonstrated that RET was among the proteins whose levels were higher in tumors with relatively low levels of NF1 binding to the KIPA beads (thus returning negative correlation coefficients, Fig. 2B; Supplementary Table S1). This was logical as the RET protein kinase is encoded by an E2-responsive gene whose expression is regulated by *ESR1* through an estrogen responsive element (ERE) (25). As predicted for an NF1-low state, NF1-low tumors also have higher levels of kinases downstream of RAS, that is, PI3Kα (encoded by *PIK3CA*) and MEK1 (encoded by *MAP2K1)*. We subsequently performed KIPA on a cohort of baseline ER^+^ tumor samples (tumor content ≥50%) accrued during a phase II trial in which patients were treated with letrozole before surgery (17). Similarly, in this pre-operative letrozole (POL) cohort, higher levels of RET and MEK1/MAP2K1 were significantly associated with NF1-low status (Fig. 2C; Supplementary Table S2).

**FIGURE 3:**
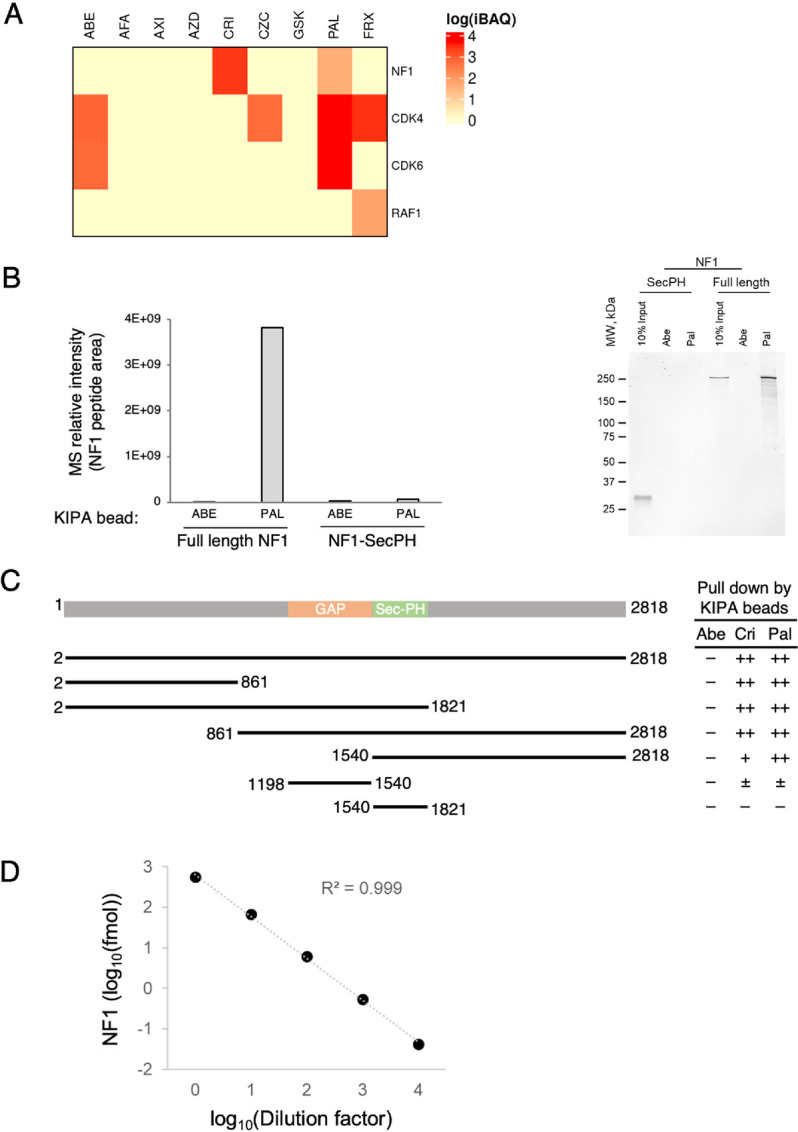
NF1 binds palbociclib-conjugated bead directly. **A,** Reference cell lysate known to contain up to 93% of kinases in human genome (see Materials and Methods; ref. 16) was incubated with beads conjugated to indicated single kinase inhibitor. A heat map was generated to show protein levels (iBAQ) on the bead as measured by MS. **B,** Purified NF1 was incubated with abemaciclib or palbociclib conjugated beads and the bound NF1 was analyzed by MS (left) or by gel electrophoresis after Flamingo staining (right). SecPH domain from NF1 was examined as the negative control. **C,** Full-length and truncated NF1 proteins were pulled down by drug-conjugated beads and analyzed by gel electrophoresis as in B. **D,** Purified NF1 (500 ng) was incubated with palbociclib beads and digested by trypsin. The products were serially diluted (1:10) before SureQuant.

We next ascertained whether the binding of NF1 to kinase inhibitor beads was drug selective. When KIPA was performed using individual kinase inhibitor beads rather than a mixture of all nine beads, we found that NF1 was only pulled down by either crizotinib- or palbociclib-conjugated beads (Fig. 3A). The latter targets CDK4/6, which were both strongly pulled down in addition to NF1 in this assay, proving a functional control. In contrast, beads that were conjugated with another approved CDK4/6 inhibitor, abemaciclib, pulled-down CDK4 and CDK6 but not NF1. Neither crizotinib- nor palbociclib-conjugated beads bound RAF. It is therefore unlikely that NF1 binds to the beads indirectly through an interaction with RAS-associated protein kinases (Fig. 3A).

Next, we investigated whether the observed KIPA interactions involving NF1 were direct by using NF1 protein and protein fragments purified from insect cells (26). We performed KIPA on purified NF1 using palbociclib-conjugated beads, while abemaciclib-conjugated beads were examined as a negative control. The pulldown samples were analyzed by either MS (Fig. 3B, left) or by gel electrophoresis followed by Flamingo fluorescent dye protein staining (Fig. 3B, right). These experiments demonstrated that purified full-length NF1, but not its SecPH domain (26), was pulled down selectively by the beads conjugated with palbociclib but not with abemaciclib. To further assess the domain of NF1 that interacts with palbociclib, we performed pulldown using additional NF1 fragments (26). The data reveal that several longer NF1 fragments also bound selectively to palbociclib (or crizotinib)-conjugated beads, suggesting that the palbociclib binding domain spans a large region in both the N- and C-termini (Fig. 3C). There was no evidence of a single drug binding pocket in NF1—both NF1_2–861_ and NF1_861–2818_ bound the drug beads almost as effectively as the full-length NF1. The GAP domain also showed weak binding, as compared with the SecPH domain, which showed no detectable binding.

To measure the amount of NF1 protein in tumor lysates quantitatively with a high degree of sensitivity, we implemented SureQuant, whereby a spiked-in heavy isotopic labeled peptide triggers high-resolution MS2 scan for a matched endogenous peptide (27). This heavy isotope peptide-guided MS acquisition provides high selectivity and sensitivity measurements of the endogenous peptides and can be used to calculate the absolute amount of protein. By spiking in 10 fmol of a heavy isotope labeled NF1 peptide in tumor lysates, it was possible to quantify NF1 with high sensitivity and linearity (Fig. 3D).

### Establishing a MS-verified IHC Assay to Detect NF1 Protein in Formalin-fixed Paraffin-embedded Tumor Samples

**FIGURE 4:**
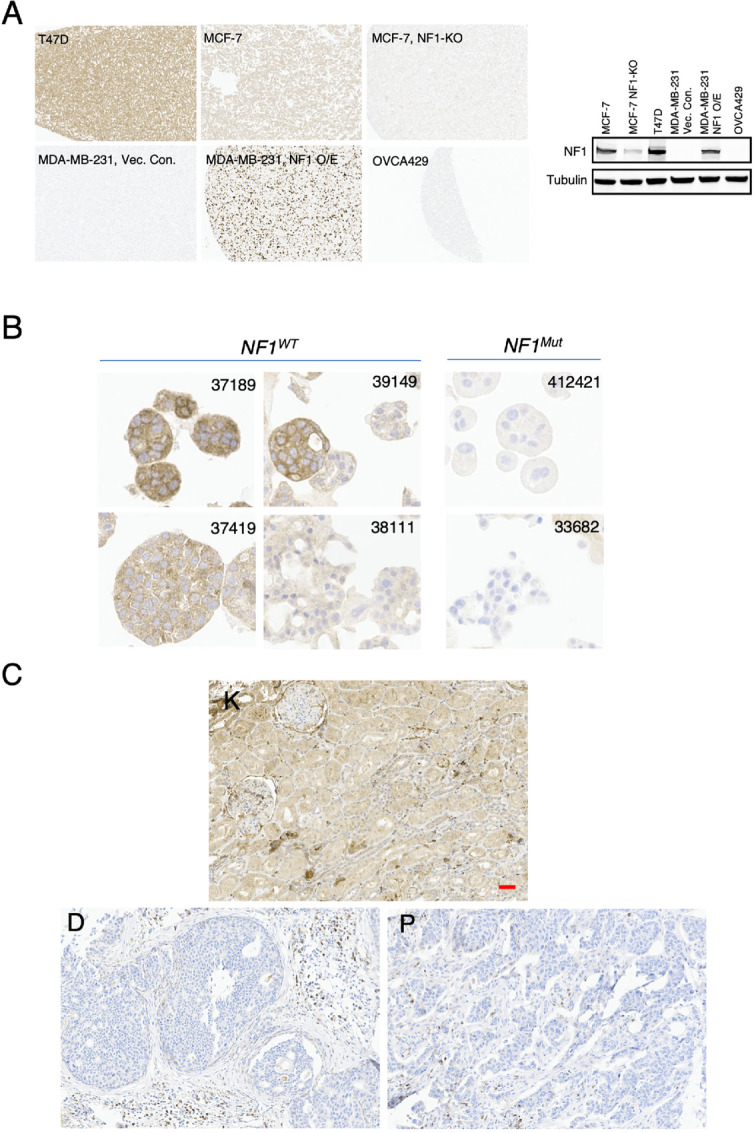
Establish an IHC assay to detect NF1 protein in FFPE tumor samples. **A,** IHC was performed on a set of cell lines with varying degrees of NF1 (left). Protein lysates from indicated cell lines were also analyzed by immunoblot as shown on the right. **B,** IHC was performed to analyze a panel of ER^+^ PDOs with known *NF1* status as determined by DNA sequencing. **C,** IHC was performed on one of the TMAs from the TMA Breast Cancer Continuum set, in which each TMA contains longitudinal samples from the same patient progressing from DCIS (**D**) to primary breast cancer (**P**). Kidney (**K**) was the normal tissue control on the TMA. Scale bar = 50 μm.

IHC is the most used method to assess protein levels in formalin-fixed and paraffin-embedded (FFPE) tumor samples in the clinic. However, a robust IHC assay to detect NF1 is not currently available. To fill this diagnostic gap, we developed a mAb that detects the C-terminus of NF1, which works well for analyzing NF1 subcellular localization by immunofluorescence microscopy (9). This antibody was used here to develop an IHC assay that accurately detected NF1 in a panel of cell lines with known NF1 levels (ref. 9; Fig. 4A). ER^+^ breast cancer cell lines T47D and MCF7 were used as positive controls. T47D cells contain twice as much NF1 protein as MCF7 cells (9). For NF1-negative controls, we included a CRISPR-mediated *NF1* knockout MCF7 line (9) and two naturally-occurring NF1-null cell lines: the ER^–^ breast cancer cell line MDA-MB-231 (9) and an ovarian cancer cell line OVCA429 (11). NF1 was reexpressed in the former by transfection to create another NF1^+^ control.

We speculated that loss of NF1 protein in tumors can be predicted by NS and FS mutations due to nonsense mRNA decay and/or protein instability. To examine this possibility using clinically relevant samples, we sought several ER^+^ PDOs models that retain the same *NF1* status as the original tumors as determined by DNA sequencing (19). The IHC data showed that while NF1 protein levels were higher in four models harboring wild-type *NF1*, NF1 levels in two *NF1* mutant models, 412421 and 33682, were much lower (Fig. 4B). Model 412421 carries NF1^S1329*/S365*^, while model 33682 carries a G to C mutation at the end of *NF1*’s intron-5, which is predicted to disrupt the 3′-splicing site leading to exon skipping.

We also carried out IHC on an ER^+^ TMA, which contains normal kidney (tissue control), ductal carcinoma *in situ* (DCIS), and primary breast tumor. Near complete loss of NF1 protein was readily detectable in the DCIS regions but not in the stroma, and this NF1-low expression state persists in locally invasive breast tumor cells (Fig. 4C).

### Near Complete Loss of NF1 Protein Predicts Treatment Responses in the PDXs

**FIGURE 5:**
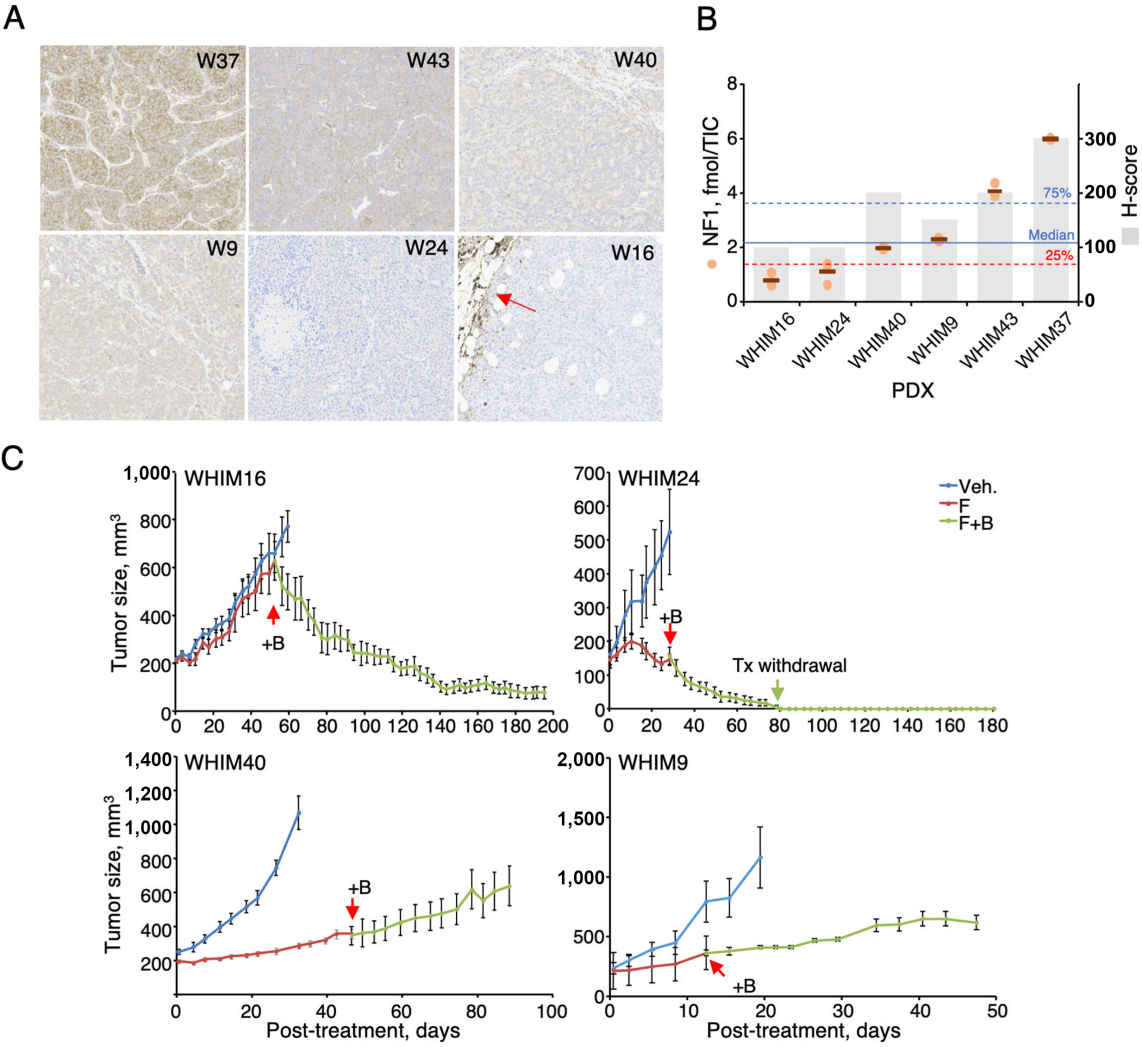
Assess the impact of NF1 protein levels on treatment responses in PDX models. **A,** IHC was performed on indicated PDX models with varying degrees of NF1 levels. **B,** Palbociclib-bead pulldown was performed on the selected PDX tumors and NF1 was quantified by SureQuant. Data were normalized by TIC (see Materials and Methods) and shown as orange circles. Median NF1 levels in this cohort is marked by a solid line, while 75% and 25% quartile NF1 levels are marked by blue and red dotted lines, respectively. H-scores (which incorporate both staining intensity and proportion into a continuous semiquantitative score ranging 0–300) for NF1 IHC are shown as light gray bars. **C,** Tumors were first treated by fulvestrant after reaching a size of 200 mm^3^ before binimetinib was later added, as marked by a red arrow (+B). Treatment withdrawn from WHIM24 was marked by a green arrow. Tumor sizes are average ± SEM. *N* = 9 mice/arm.

We have previously demonstrated that inhibition of both ER and RAS signaling by fulvestrant and binimetinib induces tumor regression in WHIM16, which was identified previously as an NF1-depleted ER^+^ PDX model as measured by RNA-seq and Western blot analysis (9). To further assess how NF1 protein levels can impact B+F treatment response, we selected three additional NF1-low models (WHIM9, WHIM24, and WHIM40) based on genomic and transcriptomic assessments (Fig. 2B). IHC (Fig. 5A; see Supplementary Table S3 for H-scores) and MS SureQuant (Fig. 5B) were performed on the same freshly harvested tumor samples, resulting in a significant positive correlation between NF1 protein levels measured using these two parallel approaches (rho = 0.88, *P* = 0.02, Spearman). These data confirmed that NF1 levels in these three models are lower than those in WHIM37 and WHIM43 (Fig 5B). As reported before, WHIM16 is a model where addition of binimetinib to fulvestrant induces tumor regression (9). In this study, we further showed that the treatment response was maintained for at least 200 days (Fig. 5C). WHIM9, WHIM24, and WHIM40 were similarly treated first with fulvestrant followed by the addition of binimetinib. These data demonstrate that WHIM24, whose NF1 levels were nearly as low as those in WHIM16, behaved similarly to WHIM16 in that tumor regressed readily after binimetinib addition to fulvestrant (Fig. 5C). Furthermore, no recurrence was observed 200 days after treatment stopped in WHIM24 (Fig. 5C). In contrast, while tumor growth delay was detected in WHIM9 and WHIM40 (Fig. 5C), no tumor regression was observed. Immunoblot analysis of tumor lysates showed substantial reduction in ER and pERK/ERK levels in these tumors, indicating the drugs were having expected on target effects (Supplementary Fig. S2).

One interpretation of the observed differential treatment responses to B+F is that NF1 protein levels are critical determinants of responsiveness to the combination. When NF1 levels fall below the detection limits of the IHC assay, as in WHIM16 and WHIM24, the tumors can respond to the treatment leading to long-term regression. By SureQuant, NF1 protein levels in WHIM16 and WHIM24 were below 25% quartile in this cohort (1.3 fmol/TIC, Fig. 5B; Supplementary Table S4). Proteogenomic analyses on the B+F resistant models were performed to more broadly examine pathway abnormalities that could negatively affect binimetinib treatment response (14, 16). WHIM9 carries an oncogenic *KRAS* mutation, as well as several other mutations affecting the RAS pathway (Supplementary Fig. S3A); furthermore, WHIM9 and WHIM40 have higher levels of MEK and ERK1 levels as compared with WHIM16 and WHIM24 (Supplementary Fig. S3B). These factors may reduce the effectiveness of binimetinib addition (see Discussion).

### Assess NF1 Protein Levels versus Treatment Response to Letrozole in Patient Tumors

**FIGURE 6:**
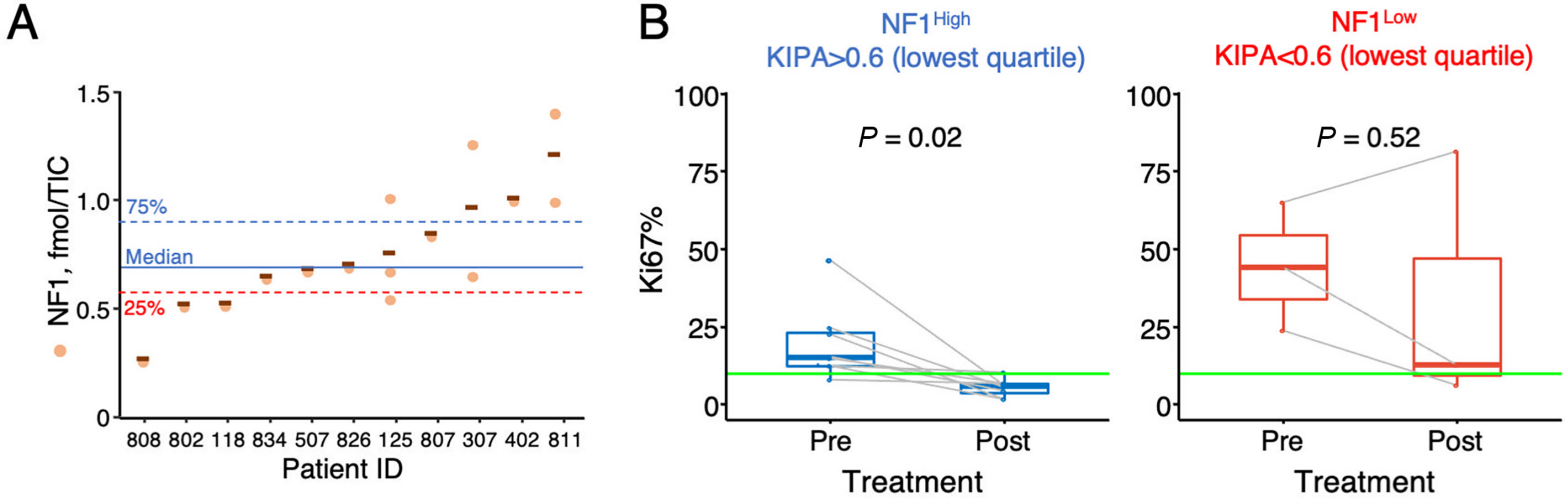
Assess the impact of NF1 protein levels on treatment responses in patient tumors. **A,** NF1 protein levels quantified by KIPA SureQuant (normalized by TIC) in each of the biopsy (orange circles) from a given POL patient were plotted and median is marked by a solid blue line. Blue and red dotted lines mark 75% and 25% quartile, respectively. The latter corresponds to 0.6 fmol/TIC. **B,** Tumors stratified by the 0.6 fmol/TIC cutoff were defined as NF1-high and NF1-low and their Ki67 levels pre-and post-letrozole treatment were plotted. *P* values were determined by paired *t* test.

Because the NCI clinical trial with B+F has not initiated enrollment, we again turned to the POL samples to provide an alternative setting to ascertain endocrine phenotype of ER^+^ tumors with respect to NF1 protein levels. Treatment response to letrozole (E2 suppression) was assessed by the proliferation marker Ki67—resistant tumors were defined as those with >10% Ki67 posttreatment (ref. 28; Supplementary Table S5). NF1 protein levels in baseline tumors (tumor content 50%–80%) were determined by KIPA-SureQuant (Supplementary Table S5; Fig. 6A). Both resistant and sensitive tumors have comparable tumor contents in the biopsy (Supplementary Fig. S4). Some, but not all, of the tumors also have FFPE samples, which were analyzed by IHC (see Supplementary Table S3 for H-scores and Supplementary Fig. S5 for all the IHC). However, no obvious correlation can be seen between the MS and IHC data, which is likely due to the small cohort size, and the fact that the MS and IHC samples are often from different biopsies. We thus proceeded with the SureQuant data because they covered more tumors and are more quantitative by design. Preclinical modeling using the PDX determined that NF1 levels at the lowest 25% quartile were an adequate cutoff to separate tumors that responded to B+F from those that did not. In the POL cohort, 25% quartile corresponds to 0.57 fmol/TIC (Fig 6A, red dotted line). Using this threshold, the tumors were classified as NF1-high and NF1-low and found that Ki67 reduction was significant in the former (Fig. 6B, *P* = 0.02 by paired *t* test). This observation suggests that tumors with adequate amounts of NF1 are sensitive to letrozole. In contrast, no significant Ki67 reduction was observed in the NF1-low group, suggesting that when NF1 fell below a threshold, the tumors would become resistant to letrozole.

## Discussion

For targeted therapy to be effective, it is essential to accurately determine the status of the biology in question. This remains a challenge in the clinical setting as the measurement of protein levels—a central arbiter of response—relies on IHC methods followed by subjective qualitative visual assessment with potentially limited dynamic range. These issues are particularly pertinent when the actionable state is low-level expression, when assessing tumor suppressor loss. In this study, guided by genomics, transcriptomics, and proteomics, we identified a panel of ER^+^ PDX models with varying degrees of NF1 expression to facilitate the development of two complementary approaches to detect NF1 protein levels. We developed an IHC assay that can be readily deployed to analyze standard FFPE samples. Furthermore, we used a novel drug bead MS SureQuant approach, which can be used to quality control the NF1 IHC or even be a stand-alone diagnostic.

Our previous preclinical modeling suggested that efficient RAS activation in ER^+^ NF1-depleted breast cancers can promote tumor cell survival when ER signaling is blocked by fulvestrant, and the consequent fulvestrant resistance can be inhibited by binimetinib (9). It is possible that when NF1 is lost in the tumors, for example, undetectable by our IHC assay or ≤1.3 fmol/TIC by MS, the growth and survival of these tumors become fully dependent on the concerted activation of both ER and RAS. This may explain why WHIM16 and WHIM24 tumors efficiently regress in the presence of both fulvestrant and binimetinib. In contrast, if tumors, for example, WHIM9 and WHIM40, have acquired additional RAS pathway alterations to resist ER inhibition, loss of NF1 is not necessary and binimetinib is ineffective. We note that similar pattern was observed in the POL patient cohort that tumors with low NF1 protein levels (<0.6 fmol/TIC) are resistant to letrozole, which agrees with our previous findings when tumors were stratified on the basis of *NF1* mRNA levels (9). Therefore, NF1 protein loss may be a clinically useful predictor of endocrine therapy resistance, a concept that should be further investigated in a larger cohort. For the POL cohort, it was much more challenging to assess NF1 protein status by IHC. Agreeing with our model that NF1-high tumors are likely to be sensitive to endocrine therapy, the two tumors with the highest IHC signals (#811 and #826) are all letrozole sensitive. Conversely, consistent with the model that NF1-low tumors are resistant to endocrine therapy, one of the three resistant tumors, #118, had no detectable IHC signal, and #802 had only medium level of NF1. Unfortunately, there was no sample available to assess NF1 by IHC in #402. However, three letrozole-sensitive tumors also had low IHC signals, #807, #307, and #834. As noted earlier, the samples for MS and IHC are often different biopsies from the same patient, and IHC quality may be further impacted by storage and fixation procedures. For our preclinical study using the PDX models, MS and IHC were performed on the same tumor sample, and all tissues were fixed soon after harvest for the same duration of time (overnight, Materials and Methods) to maintain freshness and staining uniformity.

IHC assays are subject to limited dynamic ranges, making the IHC approach challenging to assess the functional status of NF1 in tumors where NF1 levels fall between those in NF1-high versus NF1-null tumors. A MS-based approach can partly address this problem when absolute cut-off values are defined to subdivide the tumors into distinct functional categories. To realize this goal, however, a substantially large patient cohort with treatment and outcome information is needed. For example, in a study using SureQuant to define a HER2 level cutoff to predict trastuzumab response, over 200 patients were studied (29).

We note that palbociclib and abemaciclib were both designed to target the active sites of CDK4/6, and this on-target binding was confirmed in our drug bead pulldown experiments. However, in these pulldown experiments, the two drugs differ substantially in the ability to bind NF1—while palbociclib binds strongly to NF1, abemaciclib shows no detectable binding. It is therefore possible that differences in drug efficacy and toxicity between these two drugs could be explained in part by off-target binding to NF1 as well as other proteins. MS after drug bead pulldown may be a comprehensive approach to further investigate off-target interactions as these are often not routinely identified during drug development.

Despite progress in DNA sequencing technology for cancer diagnostics, our data suggest for identifying *NF1* mutations, this approach is insensitive as in many cases NF1 is lost at the protein and mRNA levels without a clear genomic lesion. The combination of IHC and MS is a practical approach to assess many therapeutic targets: while MS is highly quantitative it does not provide information on subcellular location or expression heterogeneity provided by IHC. Thus, these two approaches are highly complementary.

## Supplementary Material

Figure S1 Figure S1. NF1 gene structure and expression analyses.

Figure S2 Figure S2. Binimetinib and fulvestrant inhibited the intended targets in the two PDXs that did not regress after treatment.

Figure S3 Figure S3. Proteogenomic analyses of key biomarkers in the PDXs selected for treatment.

Figure S4 Figure S4. Tumor contents in the biopsy are comparable between the treatment sensitive and resistant groups.

Figure S5 Figure S5. IHC analysis on NF1 in baseline biopsies from the POL cohort.

Table S1 Table S1. Correlation between levels of NF1 and protein kinases in the PDXs.

Table S2 Table S2. Correlations between kinase levels as determined by KIPA and NF1 status in the POL cohort.

Table S3 Table S3. H-scores of NF1 protein levels in patient and PDX tumors as determined by IHC.

Table S4 Table S4. NF1 protein levels as measured by KIPA-SureQuant in the PDX models.

Table S5 Table S5. Ki67 and NF1 protein levels in the POL cohort.
