# Supplementary material for: Proteogenomic Approaches for the Identification of NF1/Neurofibromin-depleted Estrogen Receptor–positive Breast Cancers for Targeted Treatment
Source: Cancer Res Commun. 2023 Jul 26;3(7):1366–77. doi: 10.1158/2767-9764.CRC-23-0044 (PMC10370361; doi:10.1158/2767-9764.CRC-23-0044)
Supplement: Figure S1 — NF1 gene structure and expression analyses. [file crc-23-0044-s01.pdf]

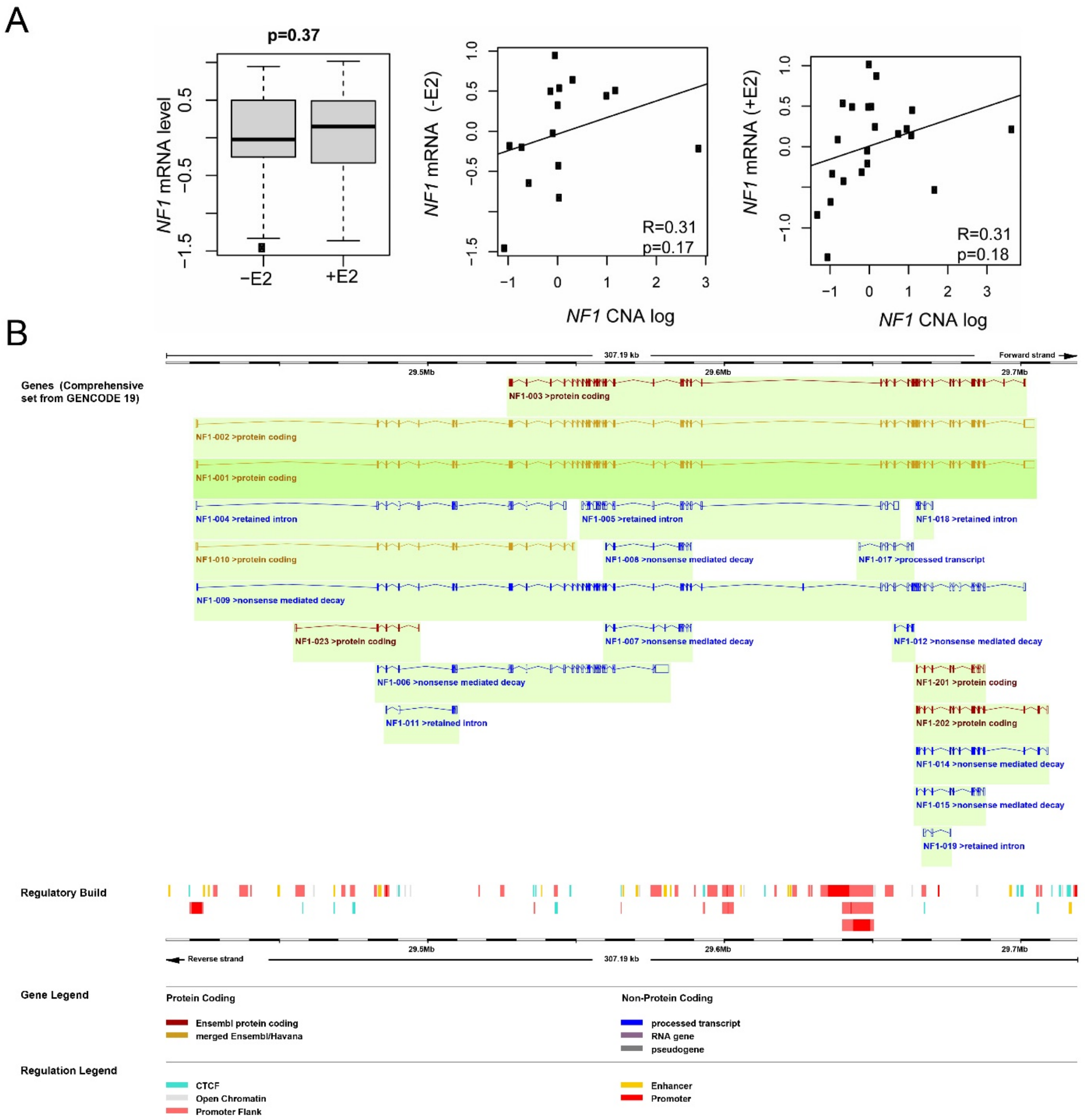

Supplementary Figure 1. *NF1* gene structure and expression analyses. (A) We have comprehensively examined gene expression in our PDX models in mice with or without E2-supplementation (Gou *et al.* (2021), see main text), and *NF1* expression was determined to be mostly un-affected by E2 (left). On the right, with or without E2, *NF1* copy numbers showed modest correlations with mRNA levels, although the results are not statistically significant. Pearson correlation coefficient (R) and p-value (p) are shown. (B) *NF1* is a very large gene with 57 consecutive exons and several alternative exons to generate a number of isoforms. We analyzed the expression from *NF1* reference sequence, NM_001042492, which encodes the longest NF1 protein by including exon 23a.
