# Supplementary material for: Proteogenomic Approaches for the Identification of NF1/Neurofibromin-depleted Estrogen Receptor–positive Breast Cancers for Targeted Treatment
Source: Cancer Res Commun. 2023 Jul 26;3(7):1366–77. doi: 10.1158/2767-9764.CRC-23-0044 (PMC10370361; doi:10.1158/2767-9764.CRC-23-0044)
Supplement: Figure S2 — Binimetinib and fulvestrant inhibited the intended targets in the two PDXs that did not regress after treatment. [file crc-23-0044-s02.pdf]

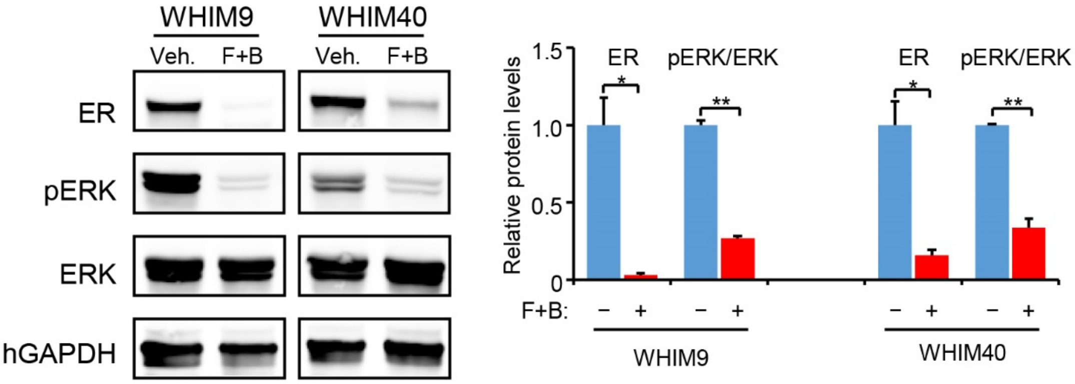


**Supplementary Figure 2.** Binimetinib and fulvestrant inhibited the intended targets in the two PDXs that did not regress after treatment. Immunoblot was performed to measure ER and pERK levels and the data were quantified on the right. p-values determined by t-test. *, $p<0.05$; **, $p<0.01$.
