# Supplementary material for: Proteogenomic Approaches for the Identification of NF1/Neurofibromin-depleted Estrogen Receptor–positive Breast Cancers for Targeted Treatment
Source: Cancer Res Commun. 2023 Jul 26;3(7):1366–77. doi: 10.1158/2767-9764.CRC-23-0044 (PMC10370361; doi:10.1158/2767-9764.CRC-23-0044)
Supplement: Figure S3 — Proteogenomic analyses of key biomarkers in the PDXs selected for treatment. [file crc-23-0044-s03.pdf]

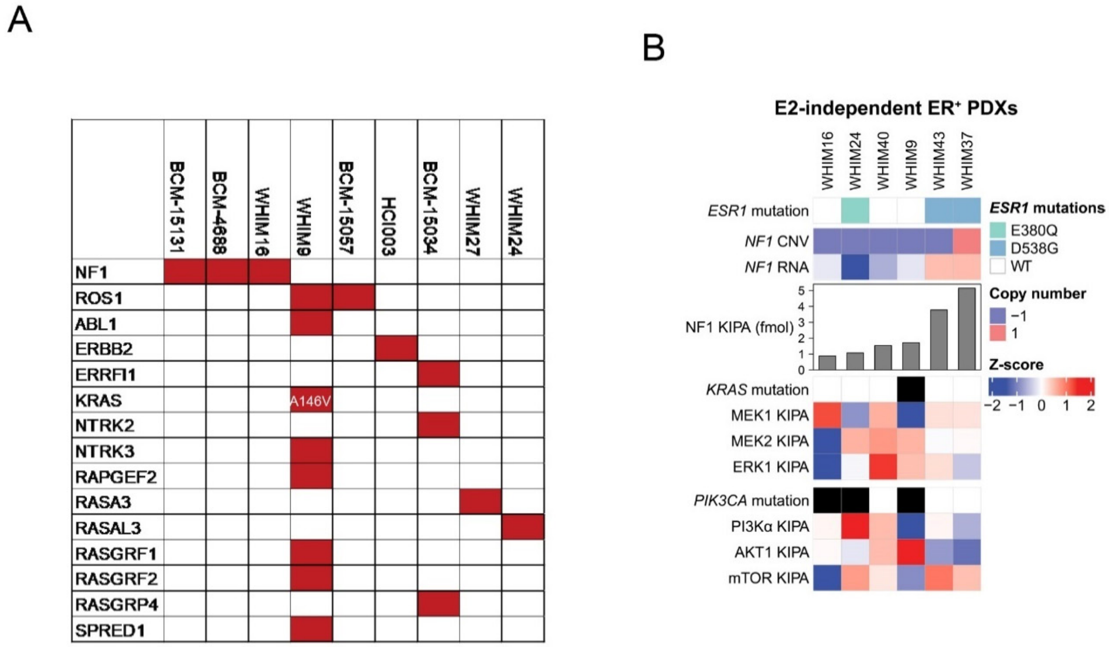

Supplementary Figure 3. Proteogenomic analyses of key biomarkers in the PDXs selected for treatment. (A) Plot showing PDX models with mutations in RAS-RTK pathway. Models (Fig. 1), such as WHIM40, without any mutations are not shown. WHIM9 has many mutations hitting the RAS pathway, including an established oncogenic *KRAS* mutation. (B) Proteogenomic data on selected PDX models are assembled in one place for comparison.
