# Supplementary material for: Proteogenomic Approaches for the Identification of NF1/Neurofibromin-depleted Estrogen Receptor–positive Breast Cancers for Targeted Treatment
Source: Cancer Res Commun. 2023 Jul 26;3(7):1366–77. doi: 10.1158/2767-9764.CRC-23-0044 (PMC10370361; doi:10.1158/2767-9764.CRC-23-0044)
Supplement: Figure S4 — Tumor contents in the biopsy are comparable between the treatment sensitive and resistant groups. [file crc-23-0044-s04.pdf]

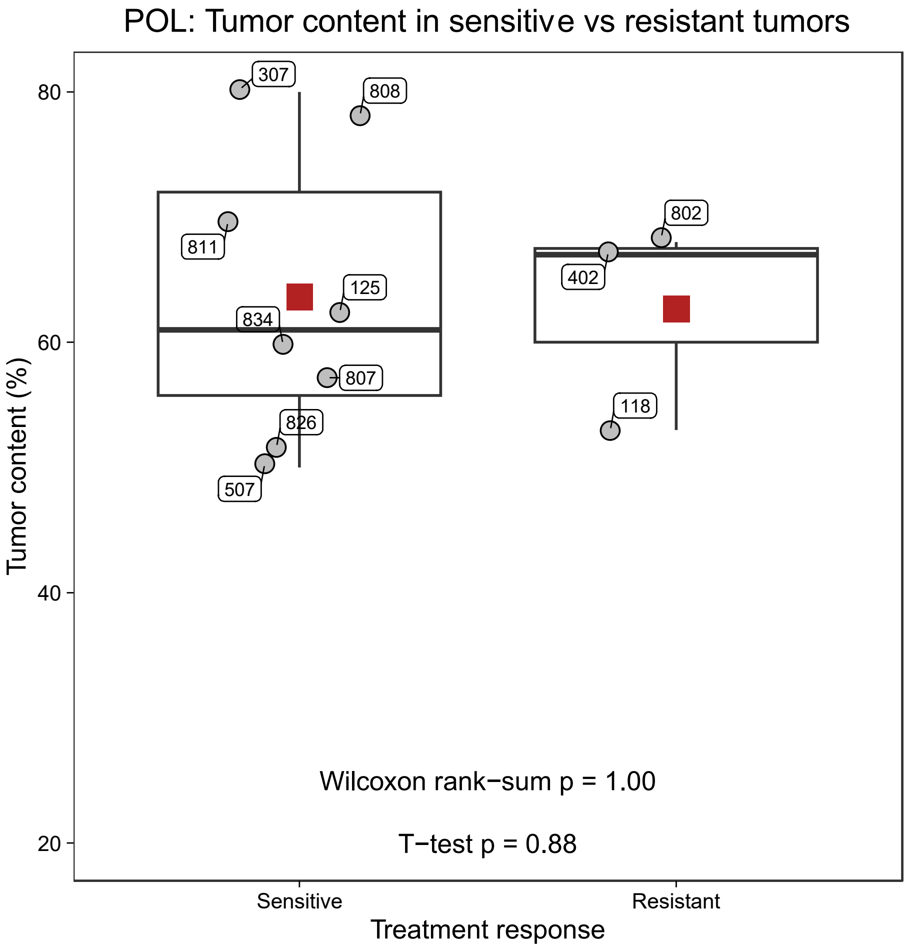

Supplemental Figure 4. Tumor contents in the biopsy are comparable between the treatment sensitive and resistant groups.
