# Supplementary material for: Proteogenomic Approaches for the Identification of NF1/Neurofibromin-depleted Estrogen Receptor–positive Breast Cancers for Targeted Treatment
Source: Cancer Res Commun. 2023 Jul 26;3(7):1366–77. doi: 10.1158/2767-9764.CRC-23-0044 (PMC10370361; doi:10.1158/2767-9764.CRC-23-0044)
Supplement: Figure S5 — IHC analysis on NF1 in baseline biopsies from the POL cohort. [file crc-23-0044-s05.pdf]

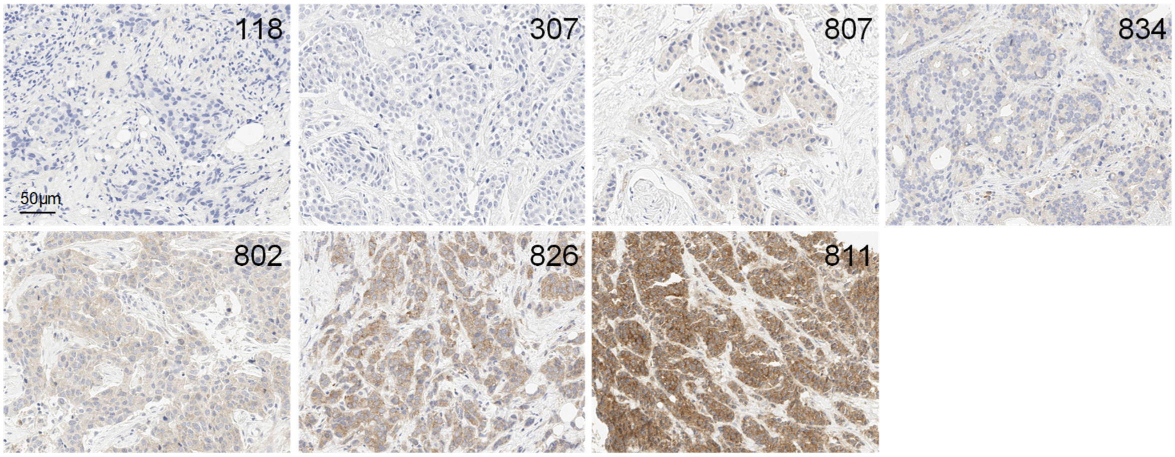

Supplementary Figure 5. IHC analysis on NF1 in baseline biopsies from the POL cohort. We note that for Patient-307, two baseline biopsies were available for MS (Fig. 6A). While one of the biopsies had high levels of NF1, the other one had low levels of NF1. There was only one baseline biopsy from Patient-307 available for IHC, as presented here, with very low NF1 levels.
